# Optimal Periods of Conducting Preventive Maintenance to Reduce Expected Downtime and Its Impact on Improving Reliability

**DOI:** 10.1155/2022/7105526

**Published:** 2022-03-03

**Authors:** Fuad S. Al-Duais, A.-B. A. Mohamed, Taghreed M. Jawa, Neveen Sayed-Ahmed

**Affiliations:** ^1^Department of Mathematics, College of Science and Humanities in Al-Aflaj, Prince Sattam Bin Abdulaziz University, Al-Kharj, Al-Aflaj 11942, Saudi Arabia; ^2^Administration Department, Administrative Science College, Thamar University, Thamar, Yemen; ^3^Department of Mathematics, Faculty of Science, Assiut University, Assiut 71515, Egypt; ^4^Department of Mathematics and Statistics, College of Science, Taif University, P.O. Box 11099, Taif 21944, Saudi Arabia

## Abstract

The present research mainly aims to use a mathematical formula to determine the optimal intervals for conducting preventive maintenance operations for machines to reduce the expected failure time when the malfunction data follow the Weibull distribution. The reliability function, failure rate, and the average time between machine failures were derived after performing preventive maintenance operations and before conducting preventive maintenance operations to state the amelioration that happens to machines. These rely on real data of performing preventive maintenance operations and the downtime required to repair machine or device faults that occur between preventive maintenance periods and the downtime necessary to perform preventive maintenance operations on the machine or device. Thus, the study concluded that preventive maintenance operations are working to increase the reliability of the machine and improve it, as well as to increase the average period of time for the machine to operate between faults.

## 1. Introduction

Because of global development, particularly in science and technology, and the use of complex and sophisticated systems, the science of reliability has many potential applications in medicine and communication areas. Further, there is an interest in the failure times and the reasons behind the sudden malfunctions that devices are exposed to lead to increased costs and decreased production which in turn lead to significant human and material losses [1–7]. The importance of the reliability deals with the probability of a particular system performing a particular job for a specific period under the working conditions for which the system is designed. By studying the reliability of the systems, the performance and efficiency of these systems can be evaluated to delineate the type and size of production and improve it through the development of engineering designs for these systems [8–11].

A decrease in reliability rates indicates a decline in the performance of the systems and the level of their efficiency. It is then necessary to find ways to improve the level of performance of these systems. Perhaps one of the most essential means in improving the status of systems and vehicles and bringing them to the desired reliability is the maintenance operation that the machines must undergo. Maintenance is vital in ensuring that the machines operate continuously with a considerable benefit behind their productive power and reducing the cost of production. So, the breakdown of equipment and machinery or being in poor condition is considered as one of the main causes of low productivity. Additionally, negligence in maintenance work is considered one of the main reasons for the high cost of production, low profit, and a major cause of waste. Many scientists dealt with determining the optimal periods for performing the preventive operations in different ways, for example, [12–19], without focusing on the effect of performing preventive maintenance operations on the reliability function, failure rate, and average time between the failures. This research aims at determining the optimal periods for carrying out preventive maintenance operations to reduce the expected time for failures. And then to explain the improvement that occurs to machines after carrying out preventive maintenance operations by calculating the reliability function, failure rate, and average time between failures of the machines before and after carrying out preventive maintenance operations. For the purpose of clarifying the sensitivity of the model with respect to the subjective values, a sensitivity analysis of the model was performed with respect to these values. The extent of the impact of those values on the optimal solution and the extent of their importance on the model were noticed by substituting different values for subjective values (the downtime required to repair faults and the downtime required to carry out preventive maintenance activities) in the model to recognize the extent of their impact on the standard periods for the purpose of carrying out the preventive maintenance operations for those machines. Then, the values were inserted in the model to recognize the extent of their impact on the standard periods for the purpose of carrying out the preventive maintenance operations for those machines.

## 2. Measuring Reliability

The indicators by which reliability can be measured are discussed below.

### 2.1. Mean Time between Failures (MTBF)

MTBF is the average time between malfunctions that occur in the vehicle or one of its repairable and irreparable parts. Thus, the higher the value of this average, the greater the availability of the vehicles, which indicates the high efficiency of the maintenance staff. MTBF can be found as follows [10]:

(1) $$\begin{array}{c} \text{MTBF}=E\left(T\right)=\;\int_{0}^{\infty }tf\left(t\right)\text{d}t\\ =\int_{0}^{\infty }R\left(t\right)\text{d}t, \end{array}$$

where *t* represents failure times, *f* (*t*) is the probability density function for failure times, and *R* (*t*) is the reliability function.

### 2.2. Mean Time to Repair (MTTR)

MTTR is the average time required to repair the vehicle after a breakdown. The higher value of this average indicates low availability of the vehicles and the low efficiency of maintenance staff.

### 2.3. Failure Rate (*h*(*t*))

The failure rate is the probability of sustaining the operation of a particular vehicle until the failure occurs. The concept of the failure rate is used to distinguish between the various distributions, and it is called the “hazard rate” in reliability studies [20].

(2) $$\begin{array}{c} h\left(t\right)=\frac{f\left(t\right)}{R\left(t\right)}. \end{array}$$

### 2.4. Availability

Availability is the ratio between the average time between faults (MTBF) to total (average time between faults plus average repair time) [21].

(3) $$\begin{array}{c} \text{Availability}=\frac{\text{MTBF}}{\text{MTBF}+\text{MTTR}}. \end{array}$$

## 3. Weibull Distribution

A study of the malfunctions and stops of specific factory machinery needs to describe the lifetimes of those machines, as expressed in a dataset representing the life span of each machine. Hence, it is preferable to use statistical distributions as models that describe lifetimes and study the reliability functions. Among the most important distributions are the exponential distribution, the Weibull distribution, the gamma distribution, the normal distribution, the logarithmic distribution, and so on. The study of the probability distribution of time to failure of any vehicle has great importance because through the failure rate, the characteristics of the machine are known.

The failure rate of any vehicle during its operational life is in two forms. The first form is a constant failure rate with time, and in this case, the machine is in its useful life stage. Therefore, the exponential distribution is the failure model that describes this stage. The second form is the time-dependent failure rate, and the Whipple distribution is one of the failure models that describe this stage.

The Weibull distribution is one of the continuous distributions commonly used in reliability studies for its ability to describe all the failure phases that a machine undergoes, such as the increasing failure phase (aging and consumption), increasing failure rate, and decreasing failure rate. The importance of distribution is highlighted through its uses in scheduling preventive maintenance activities and scheduling scrutiny [22].

The probability density function (PDF) and cumulative distribution function (CDF) of WD distribution are given as follows, respectively.

(4) $$\begin{array}{c} f\left(x\;;\;\eta ,\rho \right)=\;\frac{\rho }{\;\eta }x^{\alpha -1\;}\exp\left[-\frac{x^{\rho }}{\;\eta }\right]\;;\;x>0\eta ,\;\rho >0, \end{array}$$

(5) $$\begin{array}{c} F\left(x\;;\;\eta ,\rho \right)=1-\exp\left[-\frac{x^{\rho }}{\;\eta }\right]\;;\;x>0\eta ,\;\rho >0. \end{array}$$

The reliability function is given as follows.

(6) $$\begin{array}{c} R\left(t;\;\eta ,\rho \right)=\exp\left[-\frac{t^{\rho }}{\;\eta }\right];\;t\geq 0. \end{array}$$

Here *η* and *ρ* are scale and shape parameters, respectively.

Also, the failure rate at time *t*  is given by

(7) $$\begin{array}{c} h\left(t\eta ,\rho \right)=\frac{f\left(t;\;\eta ,\rho \right)}{R\left(t;\;\eta ,\rho \right)}=\frac{\rho }{\eta }t^{\rho -1};\;t\geq 0. \end{array}$$

The mean time between failure (MTBF) for a Weibull distribution is given by

(8) $$\begin{array}{c} \text{MTBF}=\int_{0}^{\infty }R\left(t\;;\;\eta ,\;\rho \right)\text{ d}t=\;\eta^{1/\rho }\Gamma \left(1+\frac{1}{\rho }\right). \end{array}$$

To estimate the reliability function, the hazard function, and the mean time between failures of the Weibull distribution, the maximum likelihood estimator is used as follows.

Let $\underline{x}=\;x_{1},\;x_{2},\;x_{3},\ldots x_{n}$ be the set of *n* random lifetime to the WD defined by (4); then, the likelihood function for the given sample observations is

(9) $$\begin{array}{c} L\;f\left(\underline{x};\;\eta ,\;\rho \right)=\;\prod_{i=1}^{n}\frac{\rho }{\eta }x_{i}^{\rho -1}\exp\left[-\frac{x_{i}^{\rho }}{\eta }\right]\;\\ =\;\frac{\rho^{n}}{\eta^{n}}\prod_{i=1}^{n}x_{i}^{\rho -1}\exp\left[-\;\frac{1}{\eta }\sum_{i=1}^{n}x_{i}^{\rho }\right]. \end{array}$$

The log-likelihood function of (9) is

(10) $$\begin{array}{c} \text{ln}L=n\;\ln\;\rho -n\;\ln\;\eta +\left(\rho -1\right)\sum_{i=1}^{n}\ln\;x_{i}-\frac{1}{\eta }\sum_{i=1}^{n}x_{i}^{\rho }. \end{array}$$

We differentiate (10) with respect to the unknown parameters and equal the resulting equation to zero as follows:

(11) $$\begin{array}{c} \frac{\partial \;\ln L}{\partial \;\rho }=\frac{n}{\hat{\rho }}-\frac{\sum_{i=1}^{n}x_{i}^{\hat{\rho }}\ln\;x_{i}}{\hat{\eta }}+\sum_{i=1}^{n}\ln\;x_{i}=0,\;\\ \frac{\partial \;\ln L}{\partial \;\eta }=-\frac{n}{\hat{\eta }}+\frac{\sum_{i=1}^{n}x_{i}^{\hat{\rho }}}{{\hat{\eta }}^{2}}=0. \end{array}$$

The maximum likelihood estimator of *η* is

(12) $$\begin{array}{c} {\hat{\eta }}_{ML}=\frac{\sum_{i=1}^{n}x_{i}^{\hat{\rho }}}{n}. \end{array}$$

The shape parameter ${\hat{\rho }}_{ML}$ is obtained by the Newton–Raphson method since it cannot be solved analytically.

By utilizing from the property of invariance of MLE, we can estimate *R*(*t*), *h*(*t*), and MTBF by replacing *η* and *ρ* with ${\hat{\eta }}_{ML}$ and ${\hat{\rho }}_{ML}\;$ in equations (6), (7), and (8) to obtain ${\hat{R}}_{ML}\left(t\right),{\hat{h}}_{ML}\left(t\right),$ and ${\hat{\text{BTBF}}}_{ML},$ as follows:

(13) $$\begin{array}{c} {\hat{R}}_{ML}\left(t\right)=\exp\left[-\frac{t^{{\hat{\rho }}_{ML}}}{{\hat{\eta }}_{ML}}\right], \end{array}$$

(14) $$\begin{array}{c} \hat{{\text{MTBF}}_{ML}}={\hat{\eta }}_{ML}^{1/{\hat{\rho }}_{ML}}\Gamma \left(1+\frac{1}{{\hat{\rho }}_{ML}}\right). \end{array}$$

## 4. Preventive Maintenance

It is a set of steps that are taken to prevent interruptions that result in a major loss to the company. Preventive maintenance means understanding and knowing the needs to be done through an early realization of the design status of machinery and equipment. And then the periodic checks are started, and the required measures are taken to carry out the services that include replacing spare parts, cleaning, and lubrication work. As a result, the probability of machine downtime is reduced, and the reliability of the machine is increased [23].

The preventive maintenance procedure depends on knowing the machine's failure rate by studying the failure times and analyzing them. If the failure times follow the exponential distribution, the machine is in the useful life stage. In this case, conducting a preventive maintenance operation will not reduce the possibility of the machine's malfunction, but rather it needs corrective maintenance. However, if the failure times are more and follow other distributions like a normal distribution, Weibull distribution, or other probability distributions, the machine needs to develop a scientific approach in preventive maintenance because in this case it is very useful and it reduces the possibility of sudden failure as well as reduces the downtime of the machine to a minimum. Moreover, it also leads to an increase in the efficiency of the performance of the machines.

## 5. Determining the Optimal Intervals of Preventive Maintenance

In this part of the research, the optimum periods (*K*) will be determined to carry out preventive maintenance operations to reduce the total downtime for a machine. So, this model assumes that no more than one malfunction occurs in one period of time, and in general, the expected number of faults during that period of time (*K*, 0) can be expressed as follows [24, 25]:

(15) $$\begin{array}{c} F\left(K\right)=\sum_{i=1}^{n}\left[1+F\left(K-i-1\right)\right]\int_{i}^{i+1}f\left(k\right)\text{d}k\;,\;\;K\geq 1, \end{array}$$

where *F*(*K*) indicates the expected number of malfunctions of the machine or device during the time period (0, K); *K* Indicates the length of time between one preventive maintenance and the next; and  *f*(*k*) denotes the probability density function for failure times.

As for the probability of occurrence of the first malfunction in the first period of time, it is expressed in the following formula:

(16) $$\begin{array}{c} F\left(0\right)=\int_{0}^{1}f\left(t\right)\text{d}t, \end{array}$$

whereas the total downtime per unit time during the preventive maintenance cycle is expressed as follows:

(17) $$\begin{array}{c} D\left(K\right)=\frac{F\left(K\right)K_{r}}{K+K_{p}}. \end{array}$$

Hence, *D* (*K*) represents the total downtime of a unit of time; *K*_*r*_ represents the downtime required to repair machine or device faults that occur between preventive maintenance periods; and *K*_*p*_ represents the downtime required to perform preventive maintenance operations on the machine or device.

## 6. Reliability Preventive Maintenance

The following reliability model assumes that the system has been brought back to its terms by preventive maintenance [26, 27].

Suppose that *R*_*K*_(*t*) is the reliability function of the system before preventive maintenance; *K* indicates the time period between one preventive maintenance and another; and *R*_*PM*_(*t*): represents the reliability function of the system after preventive maintenance.

The reliability function of the machine can be calculated after conducting preventive maintenance operations as follows:

(18) $$\begin{array}{c} R_{PM}\left(t\right)=R\left(t\right)\;0\leq \;t<K,\\ R_{PM}\left(t\right)=R\left(K\right)\left(R\left(t-K\right)\right)\;\;K\leq \;t<2K, \end{array}$$

where *R(t)* represents the reliability function until the first preventive maintenance is performed and *R*(*t* − *K*) represents the reliability function of overtime (*t* − *K*), as the system will return to normal operation in time *K*. Through equation (18), it is evident that the optimum periods of achieving preventive maintenance operations (*K*) have no effect on the reliability of the machine in the time period (*t* *<* *K*) while it leads to raising and improving the reliability function in the time period (*t* *>* *K*) after completing the preventive maintenance process in the period (*K*) where the cumulative effect of consumption and obsolescence in the period preceding (*K*) is absent.

Therefore, the general formula for the reliability function after performing preventive maintenance operations is

(19) $$\begin{array}{c} R_{PM}\left(t\right)=R{\left(K\right)}^{N}\left(R\left(t-NK\right)\right);\;NK\leq \;t<\left(N+1\right)K\;N=0,1,2,\ldots , \end{array}$$

where *R*(*K*)^*N*^ represents the reliability function after conducting *N* from preventive maintenance periods and *R*(*t* − *NK*) represents the reliability function of the period (*t* − *NK*) since the last preventive maintenance.

Therefore, we can write the reliability function of the Weibull distribution after performing preventive maintenance operations as follows:

(20) $$\begin{array}{c} R_{PM}\left(t\right)=\exp\left[-\frac{N\;t^{\rho }}{\eta }\right]\exp\left[-\frac{{\left(t-NK\right)}^{\rho }}{\eta }\right];\;\text{N}K\leq t<\left(N+1\right)K,\;N=0,1,2,\;\ldots . \end{array}$$

Thus, the maximum likelihood estimator of the reliability function is

(21) $$\begin{array}{c} {\hat{R}}_{PM}\left(t\right)=\exp\left[-\frac{N\;t^{{\hat{\rho }}_{ML}}}{{\hat{\eta }}_{ML}}\right]\exp\left[-\frac{{\left(t-NK\right)}^{{\hat{\rho }}_{ML}}}{{\hat{\eta }}_{ML}}\right];\;NK\leq t<\left(N+1\right)K,\;N=0,1,2. \end{array}$$

As for the estimated value of the mean time between failures (MTBF), after performing preventive maintenance operations for the Weibull distribution, it can be found after replacing *R*_*PM*_(*t*) for *R*(*t*) in (8) as follows:

(22) $$\begin{array}{c} {\text{MTBF}}_{PM}=\int_{0}^{\infty }R_{PM}\left(t\right)\text{d}t,\\ =\sum_{N=0}^{\infty }\int_{K}^{\left(N+1\right)K}R_{M}\left(t\right)\text{d}t\\ =\sum_{N=0}^{\infty }\int_{NK}^{\left(N+1\right)K}R_{M}{\left(K\right)}^{N}R\left(t-NK\right)\text{d}t. \end{array}$$

With *z*=*t* − *NK*, we find

(23) $$\begin{array}{c} {\text{MTBF}}_{M}=\sum_{N=0}^{\infty }R{\left(K\right)}^{N}\int_{0}^{T}R\left(z\right)\text{d}z, \end{array}$$

as

(24) $$\begin{array}{c} \sum_{N=0}^{\infty }R{\left(K\right)}^{N}=\frac{1}{1-R\left(k\right)}. \end{array}$$

The average time between failures after performing preventive maintenance operations will be

(25) $$\begin{array}{c} {\hat{\text{MTBF}}}_{M}=\frac{\int_{0}^{K}R\left(t\right)\text{d}t}{1-R\left(t\right)}. \end{array}$$

As for the greatest potential estimator of the failure rate (${\hat{h}}_{ML}\left(t\right)$), we can get it in terms of the reliability function as follows:

(26) $$\begin{array}{c} {\hat{h}}_{PM}\left(t\right)=\frac{\partial }{\partial t}R_{PM}\left(t\right).\frac{1}{R_{PM}\left(t\right)}. \end{array}$$

## 7. Practical Field of the Study

In this section, the optimal periods for conducting preventive maintenance (*K*) operations for the machines will be determined so that the total stopping time of the machine is the least possible based on real data representing the operating times of the machines between malfunctions. The data of two machines were taken from the machines of the National Company for the Manufacturing of Sponge and Plastic in the Republic of Yemen, considering that these times represent the hours of operation of the machines between faults for the daily meal that represents 24 working hours. Also, the faults of these machines represent mechanical faults. Moreover, the researchers took into account the maintenance times in regard to the stopping of the machines. The data were collected from the lists on which the machine's downtime was recorded, called a “shift production report.” It should be evident that the downtime required to repair faults (*K*_*r*_) and the downtime required to carry out preventive maintenance activities (*K*_*p*_) included in the model calculation are subjective values obtained from the practical experience of the maintenance engineers in the company because of the absence of documented data about these times in the company. Thus, to clarify the sensitivity of the model concerning the subjective value (*K*_*r*_, *K*_*p*_), the sensitivity analysis of the model of these values was used by substituting different values for (*K*_*r*_, *K*_*p*_) and observing the extent to which these values affect the optimal solution and their effect on the optimal periods for performing operations of the preventive maintenance (*K*) for these machines and to indicate their importance in relation to the model. Hence, the following values include the operating times of the machine during the faults in days for the studied machines (Tables1 and 2).

### 7.1. Test of Goodness of Fit

**Figure 1:**
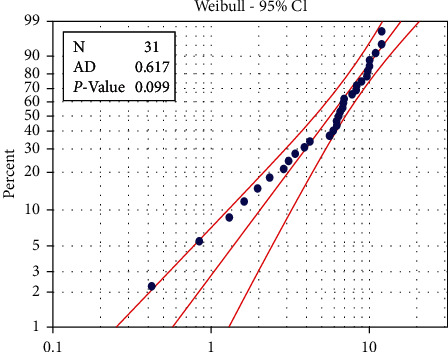
Probability plot of machine Alba26.

**Figure 2:**
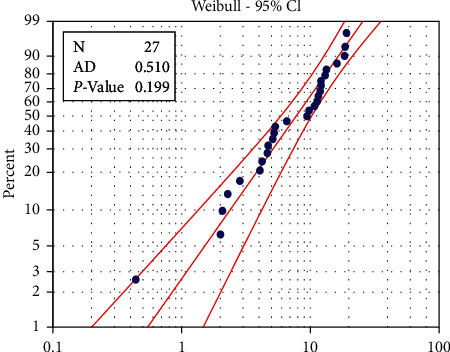
Probability plot of machine Crupp 21.

To find the suitability of the data for the proposed distribution under study, the Anderson–Darling statistical scale (AD) was used along with the value (AD) for the machine Crupp 21 (0.510). So, it was not statistically significant at the level of significance (0.05) where it reached *p* value = 0.199 < 0.05, while the value AD for the machine Alba 26 reached 0.617, so it was not statistically significant at the level of significance 0.05 where it reached *p* value = 0.199 < 0.05. Therefore, the data follow the Weibull distribution, as shown in Figures 1 and 2.

### 7.2. Results of Determining the Optimal Intervals for Performing Preventive Maintenance

The downtime required to repair faults (*K*_*r*_) and to accomplish preventive maintenance activities  (*K*_*p*_) for these machines represented by hours was (*K*_*r*_=3.75, *K*_*p*_=2.25 ); the corresponding values in days were (*K*_*r*_=0.15625, *K*_*p*_=0.09375), whereas for the optimal periods for conducting preventive maintenance operations (*K*), the expected number of faults *F(K)* and the total downtime *D(K)* were calculated as shown in Table 3.

**Figure 3:**
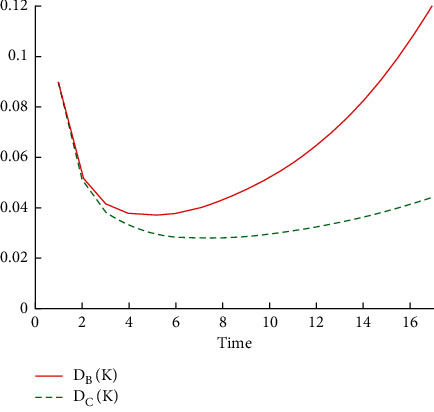
The optimal periods for performing the preventive maintenance operations and the total downtime *D* (*K*).

Through Table 3, it is clear that the optimal period for carrying out preventive maintenance operations in which the total downtime of the machine is the least possible is 5 days for the machine Alba 26, while for the machine Crupp 21 is 7 days, and the expected number from failures *F*(*K*) of Alba 26 machine increases with high rate due to the increase of the periodic time between preventive maintenance and another till it reaches approximately 13 failures within 17 days. As for the Crupp 21 machine, the expected number from failures *F*(*K*) increases due to the increase of the periodic time between preventive maintenance and another till it approximately reaches 4 failures within 17 days. As for the total stopping time *D*(*K*) for the two machines, it is noted that it gradually decreases until the optimal solution is reached and then returns to the increase, as shown in Figure 3.

Hence, to clarify the sensitivity of the model with respect to the subjective value (*K*_*r*_ ,  *K*_*p*_) for this machine, a sensitivity analysis was conducted, and the results are shown in Table 4.

Table 4 indicates that the model is insensitive concerning the values (*K*_*r*_,  *K*_*p*_). Thus, the change in these values did not affect the optimum solution (*K*) for these machines. According to the effect of preventive maintenance on the reliability function and failure rate of these machines, the reliability function and failure rate values were calculated for machines before and after they were subjected to preventive maintenance operations, and the results are shown in Table 5 for the machine Alba 26 and Table 6 for the machine Crupp 21.

Hence, the estimates of the indications of this machine were equal in days (*ρ*=1.8424, *η*=34.92219). The following table shows the values of the reliability function and the failure rate of the machine before and after it undergoes preventive maintenance.

**Figure 4:**
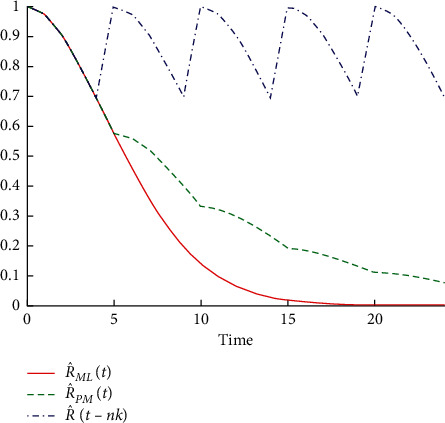
Reliability function of machine Alba 26 before and after performing preventive maintenance operations.

**Figure 5:**
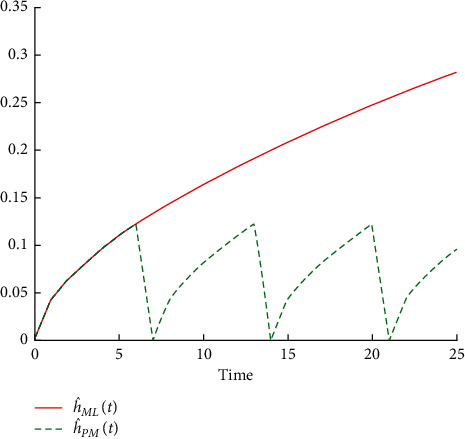
Failure rate of machine Alba 26 before and after performing preventive maintenance operations.

Table 5 shows that the reliability function decreases before and after performing the preventive maintenance operations ${\hat{R}}_{ML}\left(t\right)$ because of the rapid increase of time. So, the probability of operating this machine for 8 days without a breakdown is equal to 0.26695, and for 18 days without failure, the probability is equal to 0.00278. It continues similarly until it reaches 0.00005 when the machine operates for 24 days without a breakdown. Also, when comparing ${\hat{R}}_{ML}\left(t\right)$ with the reliability of the machine after implementing the operations of preventive maintenance ${\hat{R}}_{PM}\left(t\right)$, it decreases slowly with increasing time because of the improvement in the machine. Thus, the probability that this machine will operate for 8 days without a breakdown becomes 0.46195, that for 18 days without fault equals 0.15207, and so on until the reliability reaches 0.07499 when the machine operates for 24 days without malfunction. This is reflected by the failure rates before and after conducting the preventive maintenance operations, which return to zero value after every preventive maintenance performed. This is shown in Figures 4 and 5.

As for the average time between failures (MTBF) for this machine after completing preventive maintenance operations, it was 9.77866 days while it reached 6.87898 days before performing preventive maintenance operations.

Secondly, regarding the machine Crupp 21, the estimates of indications were equal in days to (*ρ*=1.59007, *η*=37.7744), and the following table illustrates the values of the reliability function and failure rate of the machine before and after being subjected to the preventive maintenance operations.

**Figure 6:**
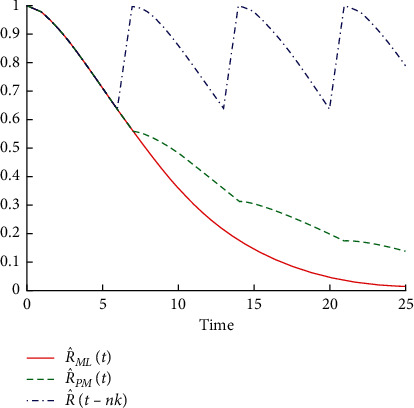
Reliability function for the machine Crupp 21 before and after performing the preventive maintenance operations.

**Figure 7:**
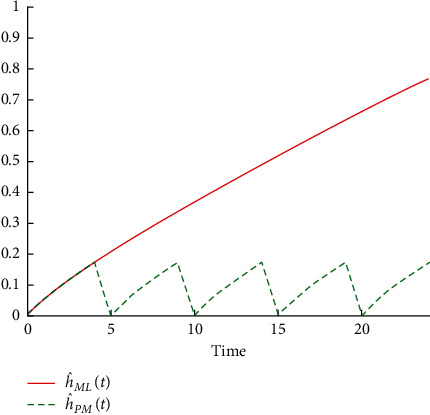
Failure rate of machine Crupp 21 before and after performing preventive maintenance operations.

Table 6 shows that the reliability function ${\hat{R}}_{ML}\left(t\right)$ before carrying out the preventive maintenance operations for the machine decreases with increasing time because the probability of operating this machine for 9 days without a breakdown is equal to 0.4185, that for 19 days without a breakdown is equal to 0.0574, and so on until the reliability reaches 0.0120 when the machine operates for 25 days without malfunction. When comparing reliability ${\hat{R}}_{ML}\left(t\right)$ with ${\hat{R}}_{PM}\left(t\right)$, which represents the reliability of the machine after accomplishing the preventive maintenance operations, it is clear that it decreased very slowly with increasing time as a result of the improvement in the machine. The probability of this machine operating for 9 days without failure is equal to 0.5148, for 19 days without an equal failure is 0.2208, and so on till the reliability reaches 0.1364 when the machine operates for 25 days without a failure. This is reflected by the failure rates before and after conducting the preventive maintenance operations which return to zero value after every preventive maintenance performed. This is shown in Figures 6 and 7.

As for the average time between failures (MTBF) for this machine after completing preventive maintenance operations, it reached 12.8167 days, whereas as for the average time between failures for this machine before the preventive maintenance was performed, it reached 8.8053 days.

## 8. Conclusion and Main Findings

1. The machines with increased failure rates and low reliability are clearly subject to preventive maintenance operations with close periods. So, the optimal periods for completing preventive maintenance operations for the Alba 26 Machine in 5 days are less than the ideal periods for completing preventive maintenance operations for the Crupp 21 machine of 7 days.
2. Applying the preventive maintenance model on the studied machines makes it evident that the preventive maintenance processes work to reduce failure rates as the rate returns to zero after performing each preventive maintenance process. Preventive maintenance works to improve the reliability of the machine as the reliability function of the Alba 26 machine before performing the first preventive maintenance was *R*(6)=0.45963, whereas it reached *R*(6)=0.55756 after conducting the preventive maintenance processes for an improvement of 21%. Likewise for Crupp 21, the reliability of the machine before carrying out the first preventive maintenance operations is R(8) = 0.4856 while it reached R(8) = 0.5430 after carrying out preventive maintenance operations, i.e., an improvement of 12%.
3. The results showed that preventive maintenance operations led to an increase in the average period of time for machines to operate between faults because the average time between failures of machine Alba 26 before the completion of preventive maintenance operations was 6.87898, whereas, after the completion of preventive maintenance, it reached 9.77866 with an increase of 42%. The same was true with the machine Crupp 21 where the percentage of increase in the average period of time for the machine to operate after the preventive maintenance process reached 46%.
4. The results concerned with estimations reliability of machines before carrying out the maintenance operations gradually decreased with the increasing time at different speeds and gave a general indication that these machines cannot be relied upon to work for long periods without failure.

The values of (*K*_*r*_, *K*_*p*_) included in the calculation of the optimal periods to accomplish preventive maintenance operations are subjective values obtained from the practical experience of maintenance engineers in the company, and when implementing the maintenance model in the company, real values of (*K*_*r*_, *K*_*p*_) will be used and entered as such on the maintenance form.

## Figures and Tables

**Table 1:** The machine Alba 26/polyethylene manufacturer.

2.3438	9.9792	8.25	3.8854	1.9583	9.6042	6.3958	5.5625	7.7708
0.8333	11.9375	5.9167	10.9375	3.0625	4.2083	6.8333	6.8854	6.2083
12.0104	8.9167	8.3125	0.4167	2.875	6.1875	6.5313	9.7708	1.2917
10.0625	3.375	6.7917	1.6042					

**Table 2:** The machine Crupp 21/injection and blowing manufacturer.

5.3958	2.0729	5.125	9.8333	19.1667	2.0104	13.1563	11.9792	18.6354
13.4271	4.6979	9.4583	2.2917	4.0625	11.3333	12.2292	18.7917	4.6667
11.5625	2.8333	10.8542	0.4375	16.2604	12.2083	5.2188	6.5938	4.2083

**Table 3:** Determining the optimal period for conducting preventive maintenance operations and total downtime *D* (*K*).

	Machine Alba 26		Machine Crupp 21	
*T*(days)	*F*(*K*)	*D*_*B*_(*K*)	*F*(*K*)	*D*_*C*_(*K*)
1	0.0286	0.0898	0.0265	0.0895
2	0.1035	0.0525	0.0804	0.0508
3	0.2218	0.0415	0.1554	0.0382
4	0.3856	0.0376	0.2503	0.0325
5	0.5992	0.0368	0.3652	0.0296
6	0.8694	0.0377	0.5012	0.0282
7	1.2052	0.0398	0.6599	0.0278
8	1.6182	0.0428	0.8434	0.0279
9	2.1224	0.0468	1.0543	0.0284
10	2.7355	0.0516	1.2956	0.0293
11	3.4787	0.0574	1.5707	0.0306
12	4.3780	0.0643	1.8838	0.0321
13	5.4649	0.0724	2.2396	0.0339
14	6.7774	0.0818	2.6433	0.0360
15	8.3612	0.0928	3.1009	0.0383
16	10.3093	0.1059	3.6303	0.0411
17	12.5770	0.1204	4.2066	0.0439

**Table 4:** The results of the sensitivity analysis for the time taken for preventive maintenance *K*_*p*_ and the used time for fault repair *K*_*r*_.

*K*_*r*_ (in hours)	*K*_*r*_ (in days)	*K*_*p*_ (in hours)	*K*_*p*_ (in days)	T (days) for Alba 26	T (days) for Crupp 21
2.5	0.10417	1.25	0.052083	5	7
3.75	0.15625	2.25	0.093750	5	7
450	0.18750	2.50	0.104167	5	7
5.25	0.21875	3.00	0.125000	5	7
6.00	0.25000	3.50	0.145833	5	7

**Table 5:** Reliability function and failure rates before and after performing preventive maintenance of the machine Alba 26.

*t* (time)	${\hat{R}}_{ML}\left(t\right)$	${\hat{R}}_{PM}\left(t\right)$	$\hat{R}\left(t-nk\right)$	${\hat{h}}_{ML}\left(t\right)$	${\hat{h}}_{PM}\left(t\right)$
0	1.00000	1.00000	1.00000	0.00000	0.00000
1	0.97177	0.97177	0.97177	0.05276	0.05276
2	0.90241	0.90241	0.90241	0.09460	0.09460
3	0.80513	0.80513	0.80513	0.13312	0.13312
4	0.69193	0.69193	0.69193	0.16963	0.16963
5	0.57376	0.57376	1.00000	0.20471	0.00000
6	0.45963	0.55756	0.97177	0.23870	0.05276
7	0.35606	0.51776	0.90241	0.27180	0.09460
8	0.26695	0.46195	0.80513	0.30416	0.13312
9	0.19383	0.39700	0.69193	0.33589	0.16963
10	0.13638	0.32920	1.00000	0.36707	0.00000
11	0.09304	0.31991	0.97177	0.39776	0.05276
12	0.06156	0.29707	0.90241	0.42801	0.09460
13	0.03953	0.26505	0.80513	0.45787	0.13312
14	0.02464	0.22778	0.69193	0.48737	0.16963
15	0.01492	0.18888	1.00000	0.51653	0.00000
16	0.00877	0.18355	0.97177	0.54540	0.05276
17	0.00501	0.17045	0.90241	0.57398	0.09460
18	0.00278	0.15207	0.80513	0.60229	0.13312
19	0.00150	0.13069	0.69193	0.63036	0.16963
20	0.00079	0.10837	1.00000	0.65820	0.00000
21	0.00040	0.10531	0.97177	0.68581	0.05276
22	0.00020	0.09780	0.90241	0.71322	0.09460
23	0.00010	0.08725	0.80513	0.74044	0.13312
24	0.00005	0.07499	0.69193	0.76747	0.16963

**Table 6:** Reliability function and failure rates before and after preventive maintenance of the machine Crupp 21.

*t* (time)	${\hat{R}}_{ML}\left(t\right)$	${\hat{R}}_{PM}\left(t\right)$	$\hat{R}\left(t-nk\right)$	${\hat{h}}_{ML}\left(t\right)$	${\hat{h}}_{PM}\left(t\right)$
0	1.0000	1.0000	1.0000	0.0000	0.0000
1	0.9739	0.9739	0.9739	0.0421	0.0421
2	0.9234	0.9234	0.9234	0.0634	0.0634
3	0.8591	0.8591	0.8591	0.0805	0.0805
4	0.7867	0.7867	0.7867	0.0954	0.0954
5	0.7102	0.7102	0.7102	0.1088	0.1088
6	0.6331	0.6331	0.6331	0.1212	0.1212
7	0.5576	0.5576	1.0000	0.1327	0.0000
8	0.4856	0.5430	0.9739	0.1436	0.0421
9	0.4185	0.5148	0.9234	0.1539	0.0634
10	0.3570	0.4790	0.8591	0.1638	0.0805
11	0.3016	0.4386	0.7867	0.1733	0.0954
12	0.2525	0.3960	0.7102	0.1824	0.1088
13	0.2094	0.3530	0.6331	0.1912	0.1212
14	0.1723	0.3109	1.0000	0.1998	0.0000
15	0.1405	0.3027	0.9739	0.2081	0.0421
16	0.1136	0.2871	0.9234	0.2161	0.0634
17	0.0912	0.2671	0.8591	0.2240	0.0805
18	0.0726	0.2445	0.7867	0.2317	0.0954
19	0.0574	0.2208	0.7102	0.2392	0.1088
20	0.0450	0.1968	0.6331	0.2466	0.1212
21	0.0350	0.1733	1.0000	0.2538	0.0000
22	0.0271	0.1688	0.9739	0.2608	0.0421
23	0.0208	0.1600	0.9234	0.2678	0.0634
24	0.0159	0.1489	0.8591	0.2746	0.0805
25	0.0120	0.1364	0.7867	0.2813	0.0954

## Data Availability

The data used to support the findings of this study are available from the corresponding author upon request.
